# Supplementary material for: High-sensitivity of initial SrO growth on the residual resistivity in epitaxial thin films of SrRuO3 on SrTiO3 (001)
Source: Sci Rep. 2021 Aug 9;11:16070. doi: 10.1038/s41598-021-95554-x (PMC8352924; doi:10.1038/s41598-021-95554-x)
Supplement: Supplementary file 1 — Supplementary Information. [file 41598_2021_95554_MOESM1_ESM.pdf]

Supplementary Sections

High-sensitivity of initial SrO growth on the residual resistivity in epitaxial thin films of $SrRuO_3$ on $SrTiO_3$(001)

Uddipta Kar, Akhilesh Kr. Singh, Song Yang, Chun-Yen Lin, Bipul Das, Chia-Hung Hsu, and Wei-Li Lee

In this supplementary information, there are four sections listed below.

1. The RHEED patterns with different initial SrO growth conditions.
2. The surface morphology, cross-sectional atomic structure, and chemical composition of the SRO films on STO.
3. The X-ray analyses of the SRO films on STO.
4. The magnetization measurements of the SRO films on STO.

1 The RHEED patterns with different initial SrO growth conditions.

Figure S1(a) shows the *in-situ* RHEED pattern of a TiO_2-terminated STO $(001)_c$ substrate at 700 °C along the $[110]_c$ direction. Figure S1(b) shows the RHEED pattern after opening the Ru shutter. Supplying Ru directly, irrespective of the Ru flux, on the TiO_2-terminated STO substrate gives the island formation. In the second test, we simultaneously opened both Sr and Ru shutters. Figure S1(c) displays the RHEED pattern of the TiO_2-terminated STO $(001)_c$ substrate at 700 °C along the $[110]_c$ direction. After opening both Sr and Ru shutters, additional spots appeared on the RHEED pattern due to the island growth as shown is Fig. S1(d). We further grew SrO on the TiO_2-terminated STO $(001)_c$ substrate for different τ_{IGD}. Figure S2(a) shows the RHHED pattern of the TiO_2-terminated STO $(001)_c$ substrate at 700 °C. Figure S2(b) illustrates the RHEED pattern for $\tau_{\mathrm{IGD}} = 66$ s $< \tau_{\mathrm{OIGD}}$ with the Sr flux of 1.69 $\times 10^{13}$ cm^{-2}s^{-1}. Figure S2(c) shows the intensity variation of the specular 00-spot and the secondary streak-lines that locates at the middle position between specular 00 and 01 spots. Figure S2(d) shows the RHEED pattern after ≈ 0.4 nm of SRO growth. Additional spots appeared, revealing an island type growth. Figure S2(e) displays the RHEED pattern after the growth of around 21.5 nm of SRO, showing the spot like features. We measured the $\rho(T)$ of this film, and a RRR of about 11.5 was found as shown in Fig. S2(f). For the condition with $\tau_{\mathrm{IGD}} > \tau_{\mathrm{OIGD}}$, we grew initial SrO layer for about 179 s with the above Sr flux. Figure S3(a) and (b) show RHEED patters of a STO substrate and before opening the Ru shutter, respectively. Secondary streak-lines are visible in Fig. S3(b). Figure S3(c) is the variation of the 00 and secondary streak-line intensity with time, where the τ_{OIGD} is indicated by vertical solid line. Figure S3(d) shows the RHEED patterns after the growth of ≈ 0.4 nm of SRO, where no additional spots were observed, and the streak-line feature remained nearly unchanged after the growth of about 21.5 nm of SRO as shown in Fig. S3(e). The

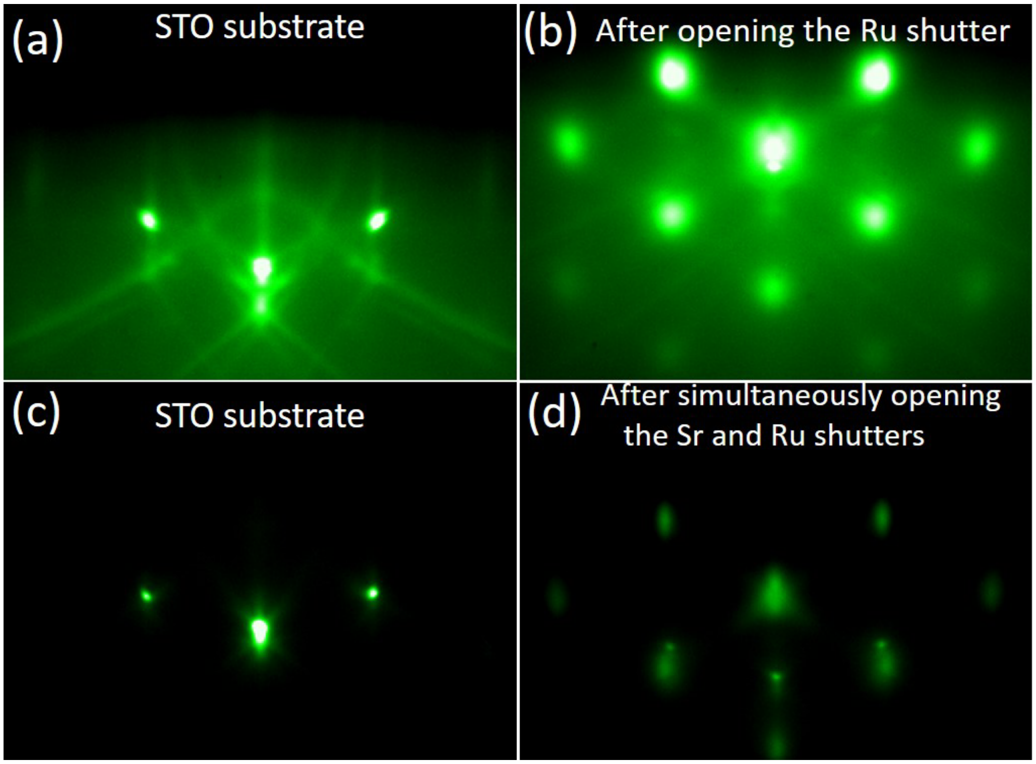

Figure S1: The RHEED patterns during SRO growth with the condition of $\tau_{\mathrm{IGD}} = 0$ s. (a) and (c) show the RHEED patterns of TiO_2-terminated STO substrates at 700 °C along STO $[110]_c$ direction. (b) RHEED pattern after supplying Ru directly on the STO substrate, showing the island type growth. (d) The RHEED pattern after opening both Sr and Ru shutters together.

corresponding temperature dependent resistivity were measured, giving a RRR of about 5.2 as shown in Fig. S3(f). Based on the above results, the condition with $\tau_{\mathrm{IGD}} = \tau_{\mathrm{OIGD}}$ was demonstrated to be the key to grow a high-quality and reproducible SRO film on STO with a low RR.

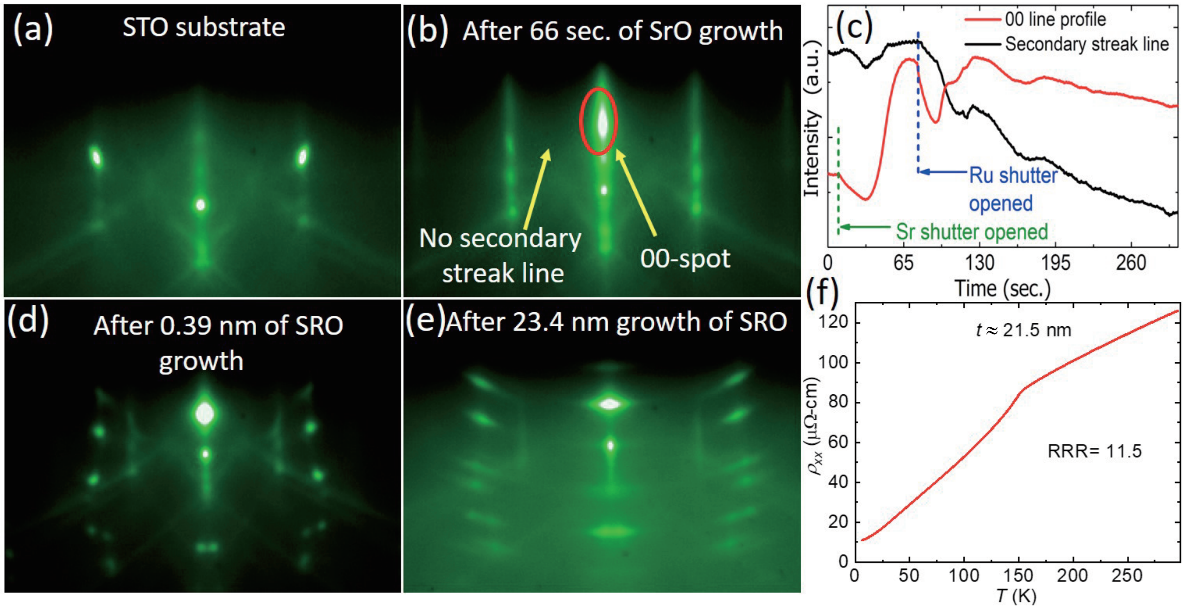

Figure S2: The RHEED patterns during SRO growth with the condition of $\tau_{\mathrm{IGD}} < \tau_{\mathrm{OIGD}}$. (a) RHEED pattern of the TiO_2-terminated STO substrates at 700 °C along STO $[110]_c$ direction. (b) RHEED pattern after $\tau_{\mathrm{IGD}} = 66$ s. (c) The RHEED intensity variation of the 00-spot and of the region between the main-streak, where the secondary streak line is expected to appear. No secondary streak-lines were observed for the above initial SrO growth duration. (d) RHEED pattern after ≈ 0.4 nm of SRO growth showing additional spots. (e) RHEED pattern after the growth of around 21.5 nm of SRO, revealing the island-type growth. (f) $\rho(T)$ curve of the above SRO film on STO.

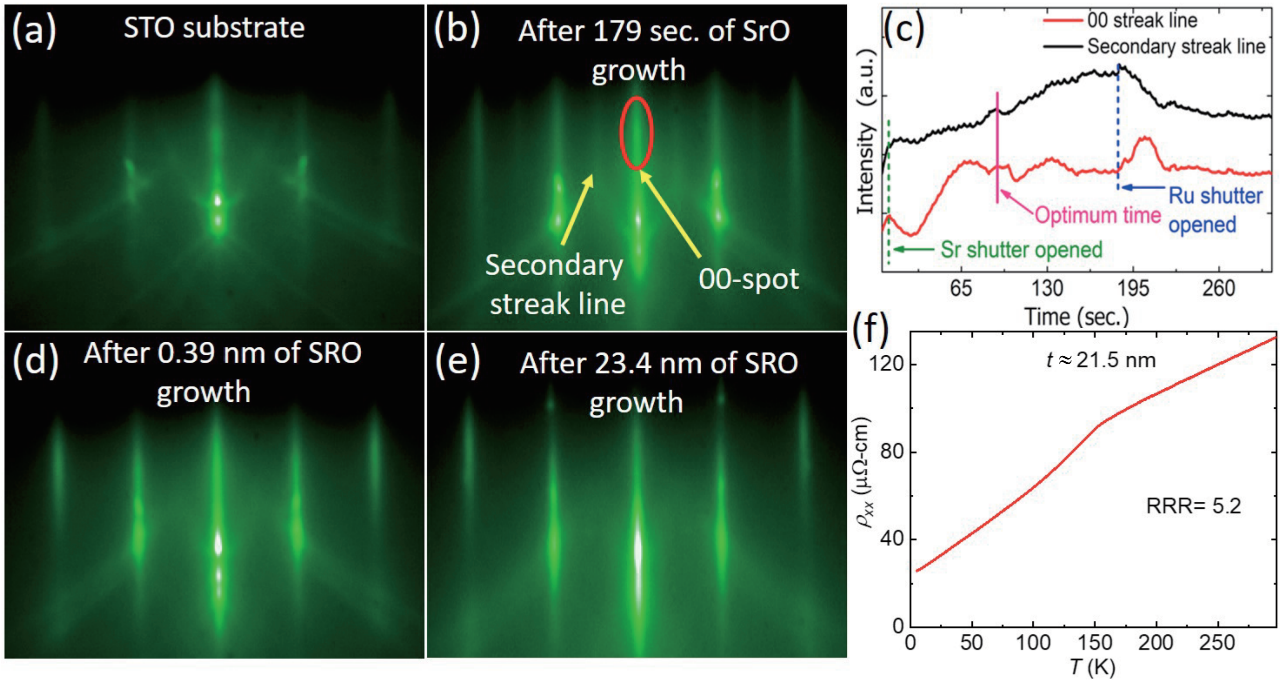

Figure S3: The RHEED patterns during SRO growth with the condition of $\tau_{\mathrm{IGD}} > \tau_{\mathrm{OIGD}}$. (a) RHEED pattern of the TiO_2-terminated STO substrates at 700 °C along STO $[110]_c$ direction. (b) RHEED pattern after $\tau_{\mathrm{IGD}} = 179$ s. Secondary streak lines appeared between the main-streak lines. (c) RHEED intensity variation of the 00-spot and one of the secondary streak line. (d) RHEED pattern after ≈ 0.4 nm of SRO growth, showing streak-line feature. (e) RHEED pattern after the growth of around 21.5 nm of SRO. (f) $\rho(T)$ curve of the above SRO film on STO.

2 The surface morphology, cross-sectional atomic structure, and chemical composition of the SRO films on STO.

Figure S4 (a) and (b) display the AFM images of a 9.0 nm thick SRO film on STO grown using the Ru flux of about 3.72×10^{13} cm^{-2}s^{-1} and 2.04×10^{13} cm^{-2}s^{-1}, respectively. When reducing the overall flux of Sr and Ru and keeping the same flux ratio, the density of surface clusters reduces significantly. In order to know the composition of the surface clusters, SEM-EDX analyses were performed on our SRO films. Figure S4(c) displays the SEM image of the SRO film grown using the above lower flux condition. Figure S4(d) shows the EDX spectra of the two regions. Region 2 is on the SRO film without any cluster, and region 1 is on the cluster as shown in Fig. S4(c). Both spectra show similar emission peak intensities for Sr, Ti and O elements, but much larger peaks for $\mathrm{Ru}_{L\alpha1}$ and $\mathrm{Ru}_{L\alpha2}$ were observed in the spectrum from surface clusters. This infers that the clusters on the film surface are most likely deriving mainly from Ru, which is further confirmed by the STEM-EDX analysis.

Fig. S5(a) displays the cross-sectional scanning transmission electron microscope (STEM) high-angle annular dark-field (HAADF) image of the SRO/STO(001) film grown with the condition of $\tau_{\mathrm{IGD}} = \tau_{\mathrm{OIGD}}$. The atomic image across the interface depicts a sharp substrate-film interface and an uniform atomic structure in the SRO film. No apparent SrO double layer was observed near the interface. On the other hand, Fig. S5(b) shows the HAADF image (upper-left) near the SRO/STO (001) interface with corresponding elementary mappings of Sr, Ru and O by STEM-EDX. Relative smooth and uniform distributions of Sr, Ru and O elements were observed in the SRO film grown with τ_{IGD} $= \tau_{\mathrm{OIGD}}$. Those results justify the sharp interface and uniform growth of SRO film on STO (001) substrate using adsorption-controlled technique with an oxide MBE system.

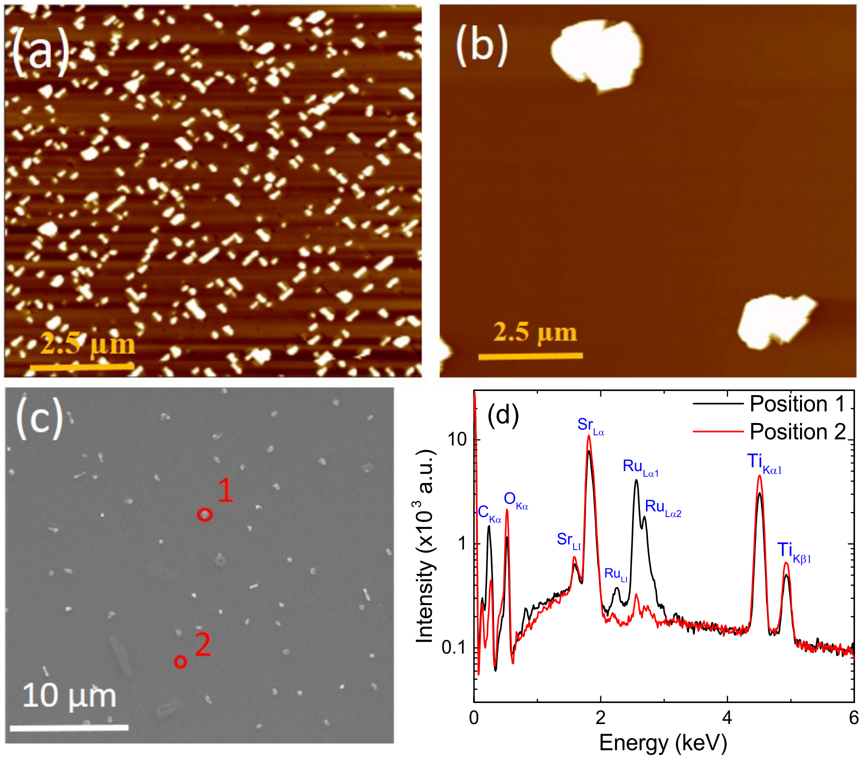

Figure S4: (a) The AFM image of a SRO film on STO grown using a Ru flux of about 3.72×10^{13} $cm^{-2}s^{-1}$ (b) The AFM image of a SRO film grown using a Ru flux of about 2.04×10^{13} $cm^{-2}s^{-1}$. (c) The SEM image of a SRO film grown using the above lower Ru flux. (d) shows the SEM-EDX spectra of a SRO film, where the probed spots for the spectrum 1 and 2 are shown in (c).

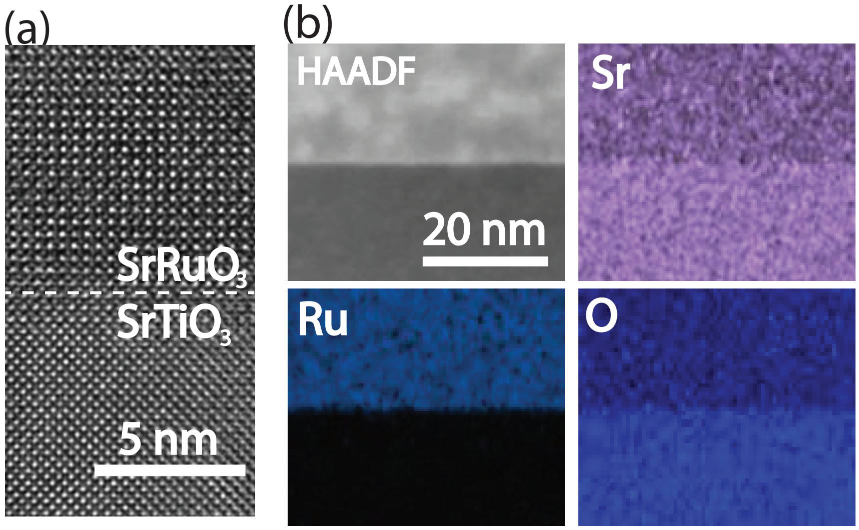

Figure S5: The cross-sectional STEM HAADF image and STEM-EDX analysis of the SRO film. (a) A cross-sectional STEM image across the SRO/STO (001) interface. (b) STEM-EDX mappings for Sr, Ru and O elements across the interface.

3 The X-ray analyses of the SRO films on STO.

Figures S6(a) and (b) display the ϕ scan across the SRO $(021)_o$ reflection for $t \approx 4.3$ nm film grown on $(\alpha, \beta) \approx$ (0.08°, 27°) and (0.56°, 1°) miscut STO substrates, respectively. Peaks originated from four rational domains, A, B, C and D, as depicted in Fig. 5(f) are well resolved. With the direciton of $\phi = 0$ assigned to align with the STO $[100]_c$ direction, the measured ϕ angles for the SRO $(02\pm1)_o$ planes from each domain are summarized in table S1, which shows excellent agreement with the observed peak locations in experiment. With increasing α and reducing β, the peak intensities for domains B, C and D were suppressed significantly, revealing that the structural domain distributions in SRO film are intimately related to the STO miscut angle and miscut direction. Figure S6(c) shows the RSMs of the STO $(204)_c$ and SRO $(260)_o$ reflections for the SRO film with $t \approx 4.3$ nm. No clear contrast in the intensity between the SRO $(260)_o$ and STO $(204)_c$ reflections was found, which gives a large uncertainty to determine the peak location for the SRO $(260)_o$ reflection. Therefore, the domain characterizations from RSM data is difficult for ultra-thin SRO films.

Figures S7(a) and (b) show the L-scans of the SRO $(211)_{o\&T}$ and SRO $(221)_o$ reflections, respectively, for SRO films with $t \approx 26.6$ and 2.7 nm. Here subscripts o and T represent the orthorhombic and tetragonal-phase, respectively. As mentioned in the main text, the SRO $(221)_o$ reflection is allowed for the orthorhombic phase but forbidden for the tetragonal phase. Whereas, the SRO $(211)_{o\&T}$ reflection is allowed for both orthorhombic phase and tetragonal phase. For the SRO film with $t \approx 26.6$ nm, pronounced peaks for both SRO $(221)_o$ and SRO $(211)_{o\&T}$ reflections were observed, reveling the orthorhombic phase. In contrast, for the SRO film with $t \approx 2.7$ nm, no detectable SRO $(221)_o$ reflection was found, while a broad peak remains observable for the SRO $(211)_{o\&T}$ reflection. This result suggests the tetragonal phase for the SRO film with $t \approx 2.7$ nm .

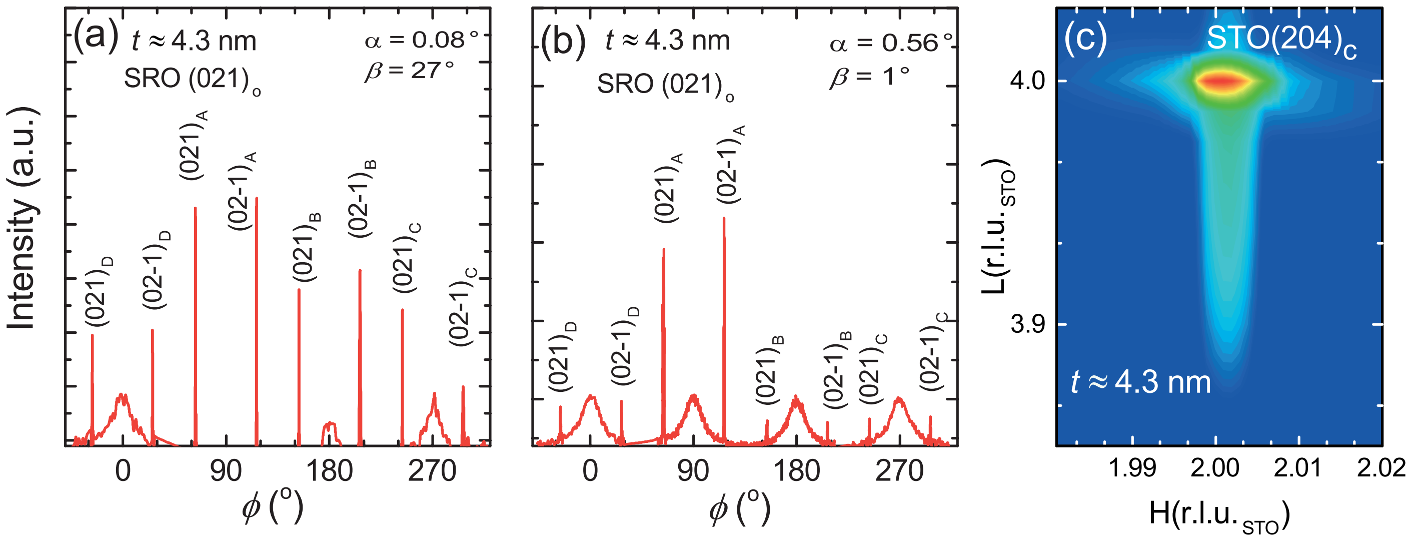

Figure S6: (a) and (b) show the azimuthal scans across the SRO $(021)_o$ reflection for SRO films grown on STO substrates with $(\alpha, \beta) \approx (0.08^o,\ 27^o)$ and $(0.56^o,\ 1^o)$, respectively. Background signals in some regions between SRO$(02\pm1)_o$ reflections in (a) were not collected due to limited experiment time. (c) shows the RSM data of a 4.3 nm thick SRO film, where intensity contrast for the SRO and STO reflections was not resolvable.

Table S1: Experimental ϕ values of SRO $(02\pm1)_o$ for the SRO film with $t \approx 26.6$ nm.

Domain	Experimental ϕ (°)
A, $[021]_o$	63.8
A, $[02\text{-}1]_o$	116.9
B, $[021]_o$	153.8
B, $[02\text{-}1]_o$	206.9
C, $[021]_o$	243.8
C, $[02\text{-}1]_o$	296.8
D, $[021]_o$	-26.5
D, $[02\text{-}1]_o$	26.8

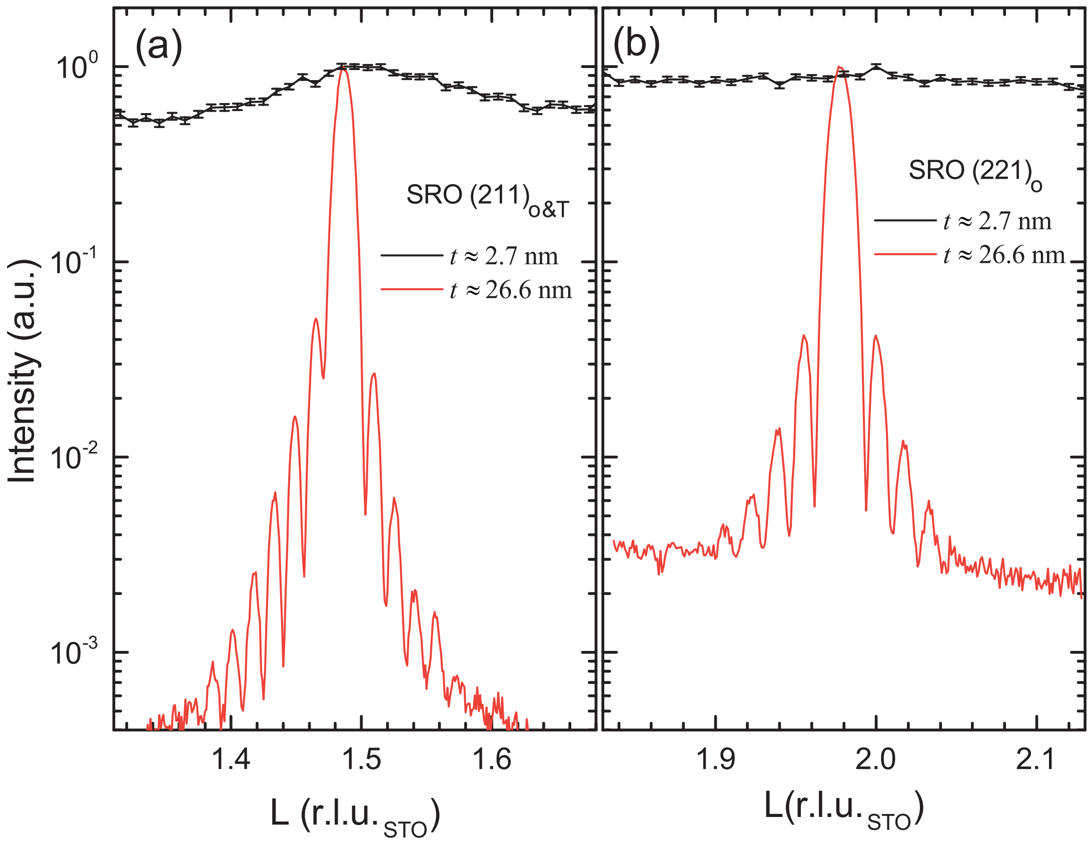

Figure S7: (a) The L-scan of the SRO $(211)_{o\&T}$ reflection, which exists in both orthorhombic and tetragonal phases. (b) the L-scans of the SRO $(221)_o$ reflection. For the SRO film with $t \approx 26.6$ nm, both SRO $(211)_{o\&T}$ and SRO $(221)_o$ are well observed, indicating an orthorhombic phase. However, for the SRO film with $t \approx 2.7$ nm, a broad peak remains observable for SRO $(211)_{o\&T}$, but the SRO$(221)_o$ reflection becomes undetectable, reveling a tetragonal phase instead.

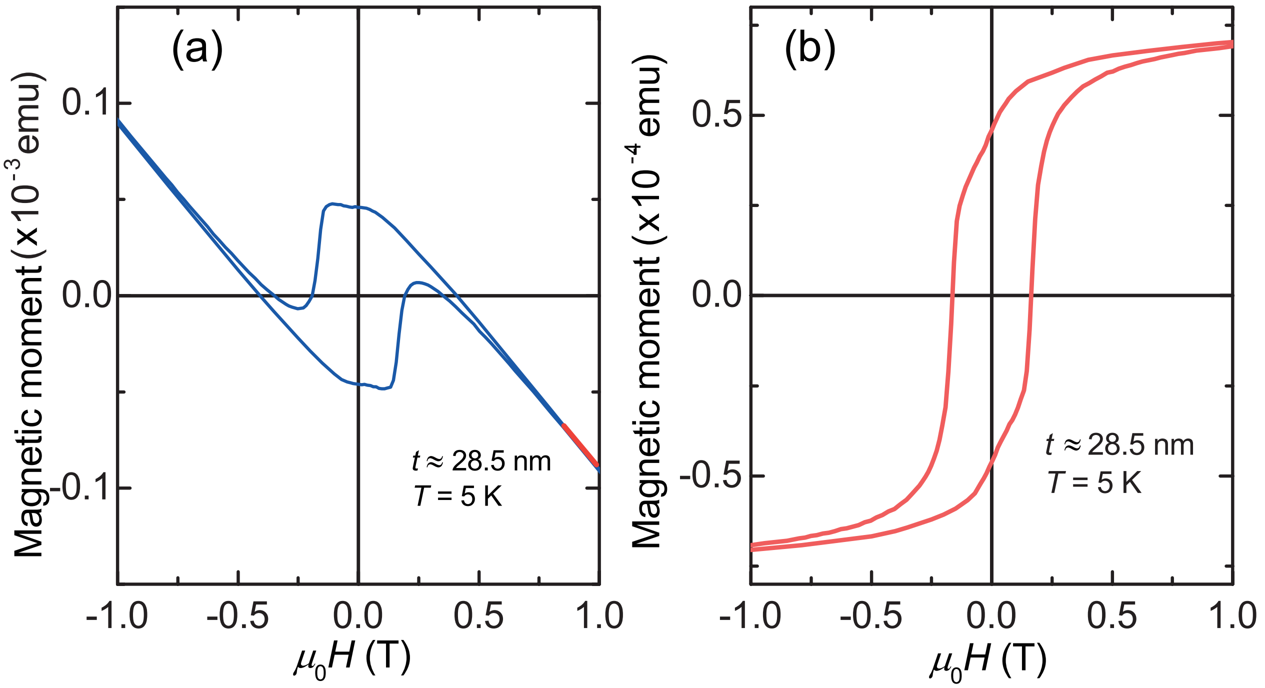

Figure S8: (a) The M-H curve of a 28.5 nm thick SRO film at 5 K. Above 0.8 T, the field dependent M data were linearly fitted and treated as the diamagnetic background from STO. (b) The M-H hysteresis loop of the SRO film after the background subtraction.

4 The magnetization measurements of the SRO films on STO.

Figure S8(a) displays the raw M-H curve for a $t \approx 28.5$ nm SRO film at 5 K. The M-H curve shows a H-linear dependent at higher field regime. The M-H data above 0.8 T were linearly fitted and treated as the diamagnetic background from STO substrate, and it was then subtracted from the M data. Figure S8(b) shows the resulting M-H curve of the SRO film after the background subtraction, and a clear hysteresis loop in M was observed in the weak field regime, revealing a ferromagnetic nature of the SRO film.
